# Characterization of *Salmonella* adaptation in response to phage treatment in broiler chickens

**DOI:** 10.1186/s13567-025-01589-7

**Published:** 2025-08-08

**Authors:** Lorna Agapé, Pierrette Menanteau, Florent Kempf, Madeline Morinet, Marianne Nicolas, Olivier Boulesteix, Mickaël Riou, Isabelle Virlogeux-Payant, Catherine Schouler, Philippe Velge

**Affiliations:** 1 INRAE, UMR ISP, Université de Tours, 37380 Nouzilly, France; 2 INRAE, PFIE, 37380 Nouzilly, France

**Keywords:** Bacteriophage, resistance, *Salmonella* Enteritidis, chicken, gut colonization, lipopolysaccharide, infection

## Abstract

*Salmonella* constitutes a significant public health threat due to its widespread association with foodborne diseases, particularly those associated with contaminated poultry products. In this context, phage therapy has emerged as a promising strategy to control these infections. However, the natural emergence of phage-insensitive bacterial strains poses challenges for the efficacy of phage therapy. Understanding the adaptive response of *Salmonella* to phages in vivo is essential for developing effective therapeutic interventions. This study investigates the adaptive responses of *Salmonella* to phages-induced challenges, deciphers the underlying mechanisms and analyzes their in vivo consequences. Following repeated administrations of a six-phage cocktail in chickens, a panel of 145 random *Salmonella* isolates was recovered and characterized. Among these, 48% exhibited reduced sensitivity to a single phage from the phage cocktail, without evidence of cross-resistance; the vast majority of isolates remained susceptible to other phages. We identified two distinct bacterial adaptation profiles both associated with modifications in the lipopolysaccharide (LPS) structure, which appears as the phage receptor. The first profile displayed a complete resistance phenotype resulting in a rough-type *Salmonella* due to a genetic mutation in the *rfbD* gene involved in LPS biosynthesis. The second profile exhibited a transient and partial resistance phenotype, due to increased LPS glucosylation, likely associated to phase variation. This phenomenon leads to coexistence of phages and bacteria within the host. Furthermore, we highlighted that these modifications could in part impair *Salmonella’s* ability to colonize the gut. Overall, our findings suggest that phage-induced evolutionary pressure may be harnessed not only to control bacterial populations but also to attenuate their pathogenicity. Therefore, bacterial resistance what is often view as a limitation of phage therapy may be leveraged as a functional advantage in phage cocktail design.

## Introduction

*Salmonella* is a major foodborne pathogen and is therefore a major public health problem worldwide [1]. The epidemiological impact of non-typhoidal serotypes is considerable. Indeed, non-typhoidal salmonellosis affects 93 million people every year worldwide, resulting in 155 000 deaths [2]. In 2023, 77 486 laboratory-confirmed cases of salmonellosis were reported in the European Union, out of which 88 were fatal – a rate of 18 cases per 100 000 population [3]. Most human cases of infection result mainly through the consumption of contaminated poultry products, such as poultry meat or eggs [4, 5]. Thus, reducing the prevalence of *Salmonella* in poultry is essential to minimizing risks to human health. However, managing contamination remains a major challenge for the poultry industry, as, depending on the countries, the use of antibiotics is either banned or discouraged due to the emergence of multidrug-resistant (MDR) strains [6, 7]. Consequently, investigation of new approaches to control *Salmonella* in poultry is crucial. Among the potential alternative approaches, the use of bacteriophages, viruses that specifically target bacteria, is emerging as a promising solution to control *Salmonella* infection in poultry [8].

Phages present real advantages over antibiotics, as individual phages target only specific bacterial strains, minimizing damage to the microbiota. Phages are easy to administer and have been granted GRAS (generally recognized as safe) status from the US Food and Drugs Administration for use as antimicrobial food additive within the food industry [9]. Moreover, they can multiply within bacterial cells, which increases their persistence thus, their therapeutic efficacy. Therefore, reports are accumulating that support the therapeutic and prophylactic use of phages to control *Salmonella* infection in poultry [8, 10, 11]. However, the success or failure of these treatments largely depends on phage delivery to poultry, phage abilities to propagate within their host in vivo and emergence of resistance to phages [12]. The latest is a key challenge in phage therapy as bacterial adaptation to an individual phage is an inevitable outcome in response to phage exposure. Thus, understanding underlying mechanisms of phage resistance is essential for developing effective therapeutic strategies. Although resistance acquisition has been reported in some studies with recovery of phage-resistant clones [13], only few studies have investigated the characterization of phage resistance in in vivo trials, which is more complex but crucial for managing resistance and for successful phage efficacy in vivo [14].

The defense mechanisms established by bacteria to cope with phage infection can be related to various strategies, such as preventing entry of phage DNA or cleaving it. These mechanisms include super-infection exclusion systems, abortive infection systems, restriction-modification systems, and CRISPR-Cas systems [15]. Additionally, bacteria can employ strategies to hinder phage adsorption, such as the production of competitive inhibitors or extracellular matrix, as well as mutations in phage receptors, all leading to phenotypic variations [16]. For example, modifying bacterial surface elements such as lipopolysaccharides (LPS), outer membrane proteins (OMPs), teichoic acids, capsular polysaccharides, flagella, or pili, is a common defense strategy employed by bacteria to prevent attachment and entry of phage genetic material into the bacteria [17]. Combining several phages that target the same bacteria in a cocktail could mitigate the development of phage-resistant strains [18].

We previously evaluated the efficacy of a cocktail of six phages as a preventive measure and demonstrated its ability to reduce the *Salmonella* load during the early stages of poultry production [11]. However, the efficacy of the phage treatment was affected by the availability of phages during infection and their persistence in the animal. In order to increase the long-term efficacy of phages, this study evaluated the repeated administration of phages to control *Salmonella* infection in chickens and analyzed the consequences on induction of bacterial resistance. Furthermore, we investigated the costs of this adaptation on the ability of *Salmonella* to colonize the gut in vivo.

## Materials and methods

### Ethics statement

In vivo experimental infections were performed at the PFIE (UE-1277 PFIE, INRAE Centre de Recherche Val de Loire, France). The experiments with chickens were carried out in strict accordance with French legislation. All animal care and use adhered to French animal welfare laws. The protocols for this study were approved by Loire Valley ethical review board (CEEA VdL, committee number 19) and the French Ministry of Education, Higher Education and Research (Ministère de l’Éducation Nationale, de l’Enseignement Supérieur et de la Recherche) under the protocol N° APAFIS#24688–202009081211981 v1. The principles of reduction, replacement and refinement were implemented in all the experiments.

### Bacterial strain and phages used in this study

The *Salmonella* Enteritidis (*S*E) LA5 strain [19] (a nalidixic acid resistant and a spontaneous streptomycin resistant strain) was isolated from broiler chicken and is characterized in the studies of Grepinet et al., [20] and Dibb-Fuller et al., [21]. The six lytic bacteriophages (SalE_1, SalE_2, SalE_3, SalE_4, SalE_5, SalE_6) used in this study were isolated from environmental water and their specificity to *Salmonella* was previously described [11]. The different phages, generally recognized as safe (GRAS) by the FDA, were supplied by a commercial entity which originally commercialized phages for food surface decontamination. For liquid cultures, bacteria preserved at −80 °C in 50% (v/v) glycerol, were routinely cultivated in lysogeny broth (LB, Miller formula) and grown overnight at 37 °C at 180 rpm. The specificity of the phages was previously described by testing them against over 1100 *Salmonella* strains of different serotypes. All phages with the exception of SalE_1, have a lytic activity against the LA5 *S.* Enteritidis strain used in this study.

### Bacterial and phage titration

*Salmonella* titration methodology from in vivo samples and phage titration methodology used in this study were previously described [11]. Briefly, *Salmonella* titration from in vivo samples was performed on crushed organ and homogenized with a bagmixer (MiniMix CC). Suspensions were then decimally diluted in Dulbecco phosphate-buffered saline with Ca2 + and Mg2 + (DPBS) (Thermo Fisher Scientific, USA) and were plated onto selective *Salmonella-Shigella* (*SS*) agar medium (Bio rad, France), supplemented with 500 µg/mL of streptomycin.

Phage titers were determined by spot assays. Serial tenfold dilutions of phages in DPBS were spotted on double agar overlay with lawns of the *Salmonella* strain of interest in molten soft 0.5% (w/v) LB agarose (10 mM MgSO_4_, 1 mM CaCl_2_ and 30 µM 2,3,5-triphenyltetrazolium chloride) poured onto 15% (w/v) LB agar plates as previously described [22].

Plates were incubated overnight at 37 °C before enumeration of *Salmonella* colonies or phages. *Salmonella* titer was expressed as CFU/g of organs. Phage titers were expressed as PFU/mL.

### Detection of bacteriophage-insensitive *Salmonella* clones

*Salmonella* clones isolated from chicken were tested for phage susceptibility using a spot assay, as previously described [22]. Briefly, *Salmonella* clones isolated from chicken cecal contents were grown in subcultures, plated to form confluent bacterial lawns. Serial tenfold dilutions of the six individual phages were then spotted onto these lawns. Following incubation, plates were visually inspected for zones of lysis. A clear zone of lysis indicated phage susceptibility, reduction of lysis (reduced plating efficiency) indicated reduced phage susceptibility, hazy or turbid zone of lysis indicated partial phage resistance and lack of lysis indicated complete phage resistance (Figure 2D). A total of 145 *Salmonella* isolates, randomly recovered from the ceca of various animals in the present study were tested. To ensure a good representation we selected 5 isolates from 29 different chickens at different time points as described Figure 2A. Additionally, 22 *Salmonella* isolates from cecal contents of chicks belonging to the independent trial [11] were included (Figure 2B). Both the present study and the independent trial were performed with the same phage cocktail and the same *Salmonella* challenge strain to ensure consistency across experiments.

### Stability of the different resistance phenotypes

To evaluate the stability of the observed resistance phenotypes, isolates recovered from chicken cecal contents were randomly selected, two identified as partially resistant and one identified as fully resistant (assigned as ΔSE10, ΔSE17 and ΔSE22 respectively), then tested for sensitivity. These selected isolates were subjected to re-isolation by streaking onto LB plates and obtained colonies (daughter cells) were inoculated into 5 mL of LB medium. The inoculated media were then incubated overnight at 37 °C, 180 rpm. Then, 100 µL of the bacterial cultures were subcultured in 5 mL LB medium and incubated at 37 °C and 180 rpm for 2 h. These bacterial solutions were being tested for sensitivity using phage spot test as described in the section above. Three-consecutive colony isolations and subcultures were performed with daughter cells recovered at each stage of consecutive colony isolations, in order to characterize the stability of the resistance phenotypes. Five randomly clones from these subcultures were studied in greater detail. Three isolates from different animals were subcultured in vitro: two partially resistant clones (ΔSE10 and ΔSE17) and one resistant clone (ΔSE22). The 5 clones (ΔSE10-1–2, ΔSE17-2, ΔSE10-1, ΔSE17-1, ΔSE22) further analyzed were derived from these subcultures, and their parentage is described in Figure 3.

### Phage adsorption and one-step growth

To understand whether the observed phage resistance phenotypes are due to a reduced capacity of the phage to adsorb on and multiply in the *Salmonella* strains, adsorption rate, burst size and latent period were determined, with one step growth curves, on the five selected isolated clones. For these experiments, subcultures of *Salmonella* bacterial clones in exponential phase in LB medium (10 mM MgSO_4_, 1 mM CaCl_2_) were adjusted to yield a cell density of 10^7^ CFU/mL and phages were added to reach a multiplicity of infection (MOI) of 0.1 (Phages_added_).

To determine the adsorption rate, after 15 min incubation at 37 °C, a 100 µL sample was collected and centrifuged at 13 000 rpm for 30 s, as previously described [23]. The supernatant was used for free phage quantification as PFU/mL using the spot assay method (Phages_supernatant_). The percentage of adsorption was estimated by the formula [(Phages_added_ – Phages_supernatant_)/ Phages_added_] × 100%. Tree independent experiments were performed.

To perform one-step growth curves, phage adsorption was allowed to proceed for 10 min at 37 °C. Adsorption was then halted by a 100-fold dilution, minimizing the impact of free phages in the culture medium. Following this synchronization step, 100 µL aliquots were collected from the static culture every 5 min for 55 min for phage quantification as PFU/mL using the spot assay method [22]. Latent period was estimated as the time between adsorption and cell lysis (phage release) and burst size was estimated by calculating the ratio of liberated phages to PFU enumerated prior to the onset of lysis (Phages_added_) [23]. Tree independent experiments were performed for each phage test.

### Auto-aggregation assays

The auto-aggregation assay performed were based on the method previously described by Zhou et al., with slight modifications [24]. Briefly, liquid cultures of bacteria were grown for 18 h at 37 °C statically in 5 mL of lysogeny broth (LB, Miller formula). The optical density (OD_600_) of the upper part of the culture (0.5 mL) was measured and recorded as OD_supernatant_. The remaining culture was then re-suspended by vortexing and the optical density (OD_600_) of 0.5 mL of the suspension was measured and recorded as OD_suspended_. The percentage rate of aggregation was estimated by the formula [(OD_suspended_ – OD_supernatant_)/ OD_suspended_] × 100%. Tree independent experiments were performed.

### LPS analysis of resistant and partially resistant clones

The LPS profiles of the 5 selected clones were investigated according to the method developed by Hitchcock et al. [25] as described in the protocol of Védrine et al. [26] with slight modifications. Briefly, bacterial cultures were grown overnight in LB medium and then collected by centrifugation at 13 000 × *g* for 10 min. The pellet was suspended in 0.0625 M Tris buffer (pH 6.8) to a concentration equivalent to 2.10^8^ CFU/mL, adjusted by optical density, then 20 µL of the bacterial culture was combined with 5 µL of lysis buffer (1% w/v SDS, 0.0625 M Tris buffer (pH 6.8)). The samples were incubated at 100 °C for 10 min then cooled to room temperature. Ten microliters of 10 mg/mL proteinase K solution (Sigma) were added per 25 μL of sample and incubated at 55 °C for 2 h, then kept overnight at room temperature. Five microliters of 4X Laemmli Sample Buffer (Bio Rad, France) were added to the samples that were subsequently incubated at 100 °C for 10 min. The obtained lysates were loaded onto a 14 × 14 cm 15% polyacrylamide gel and a 4% stacking gel and electrophoresis was performed at 30 mA current in Tris–glycine-SDS buffer (Bio Rad, France) at 17 °C. LPS profiles were revealed by silver staining after periodate oxidation as described previously [27].

The LPS composition of the 5 selected clones were then investigated. Bacteria in exponential growth phase in LB medium were inactivated using a solution of 1% phenol and extraction was performed for 20 min. at 68 °C by stirring gently. The collected aqueous and phenol phases were dialyzed against water, for 4 days, (12 0000–14 000 MWCO) to remove residual phenol. LPS was then digested enzymatically with Benzonase for 18 h at 37 °C. Proteinase K (50 µg/mL) was added and the mixture was incubated for 1 h at 50 °C. After one night at 37 °C, the samples were dialyzed against water (12–14 kDa MWCO) at 4 °C, followed by centrifugation at 100 000 × *g* for 6 h at 4 °C to precipitate the LPS. The polysaccharide portion of LPS was released from lipid A by mild hydrolysis in the presence of 1% HOAc for 2 h at 100 °C and purified by threefold extraction with chloroform. Glycosyl Composition, Linkage, and NMR analysis of O-specific polysaccharides were performed by the Complex Carbohydrate Research Center, (Univ. Georgia). Glycosyl Composition analysis was performed by combined gas chromatography-mass spectrometry (GC–MS) of the O-trimethylsilyl (TMS) methyl glycoside derivatives according to Santander et al. [28]. Glycosyl linkage analysis was performed by combined gas chromatography-mass spectrometry (GC–MS) of the partially methylated alditol acetates (PMAAs) derivatives produced form the samples as determined previously [29].

### Whole genome sequencing

The five selected isolated clones underwent whole genome sequencing to determine potential genetic changes. Bacterial genomic DNA was first extracted using the Macherey Nagel’s NucleoSpin^®^ DNA Stool kit (MACHEREY–NAGEL, Germany) following the manufacturer’s instructions with slight modifications. Briefly, after the sample preparation step, a mechanical lysis step was added with the Precellys Evolution instrument with activated Cryolys (Bertin technologies, France) for 90 s at 9000 rpm and 4 °C. The samples were then further lysed by adding 20 µL of 10 mg/mL proteinase K solution (Sigma) and incubated 30 min at 70 °C. Nanodrop One (Thermo Scientific^™^, France) was used to quantify DNA samples and agarose gel electrophoresis was performed to verify the quality of the samples. The genomic DNA obtained was sequenced by Illumina in short reads using the NovaSeq platform, PE150 (Novogene, UK) and using paired end 2 × 350 bp cycles and also sequenced by long-read using the MinION Oxford Nanopore technology (Oxford NANOPORE Technologies) following the Rapid sequencing DNA—PCR Barcoding protocol (SQK-RPB004), to achieve better resolution of sequencing. The reference strain *Salmonella* Enteritidis LA5 was subjected to the same sequencing procedure and the complete genome was obtained by hybrid assembly of Illumina and Nanopore sequences. Hybrid genome assembly was performed using the Unicycler V0.4.7 pipeline [30] with SPAdes first to assemble the Illumina reads. Nanopore sequences were filtered using nanofilt v.2.5.0 [31] to keep sequences > 500 pb and with quality phred score ≥ 7. The Illumina reads were cleaned using Trimmomatic tool V0.38 [32] before hybrid assembly. The genome was annotated by the MicroScope platform (Microbial Genome Annotation & Analysis Platform, Genoscope French National Sequencing Center) [33].

### Mutation identification

The different genomes obtained were aligned by mapping to the reference genome (genome of *S*E LA5) and compared to detect single nucleotide polymorphisms, deletions or insertions using Snippy v4.6.0. All bacterial mutations were further investigated and visualized using Geneious 10.2.6 software.

In addition to the five sequenced clones derived from three different animals, 17 additional clones, obtained from animals involved in two independent in vivo trials evaluating the effect of phage treatment (the present study and another in vivo experiment already published [11]), were included in the analysis. These clones had been previously classified as partially resistant (*n* = 13) or resistant (*n* = 4). All were then screened for the presence of mutations previously identified in the *rfbD*, *motB*, *nlpD* genes using PCR assays (primers listed in Table 1), followed by Sanger sequencing. Briefly, PCR products were obtained using the Fidelio Hot Start PCR kit (Ozyme, France) and comprised 10 µM of each primer, 0.2 mM deoxynucleoside triphosphates, 2 U of Fidelio^®^ Hot Start DNA Polymerase enzyme, 1 × Fidelio^®^ HF Buffer and 5 μL of the DNA templates. The PCR was performed for 30 cycles as follows: denaturation at 98 °C for 10 s, annealing at 65 °C for 30 s, extension at 72 °C for 30 s. An additional initial denaturation step at 98 °C for 4 min and a final step of extension at 72 °C for 5 min were performed. The PCR products were separated on a 2% agarose gel in TAE buffer (Thermo Scientific™, France) and stained with Midori Green (NIPPON Genetics EUROPE, Germany) to visualized fragments. PCR products were then purified using the NucleoSpin^®^ Gel and PCR clean-up kit (MACHEREY–NAGEL, Germany) according to manufacturer’s protocol and subjected to Sanger sequencing performed by Azenta/GENEWIZ (Azenta Life Science, Germany).

**Table 1:** **Primer sequences of genes carrying the identified mutations**

Genes	Primers	Primer sequences (5’-3’)	PCR product size (pb)
*motB*	Forward	CTATCACCTCGGTTCCGCTTTT	921
	Reverse	GCTCATCCCATTGTCGTCGTAA	
*nlpD*	Forward	CTATACGGGTGGCAGTACTTACAC	656
	Reverse	GGACCAGCATCGTATCATTATGGG	
*rfbD*	Forward	GGCACCAGTAGGGAATCTGATTG	906
	Reverse	GCCCTTACGCGTTTTCATTTCC	

### Experimental design of in vivo trials

The two trials were performed using commercial Ross 308 broiler chickens. Fertilized eggs were obtained from the incubation establishment BOYE Accouvage (France) with hatchings proceeded in confined conditions at the experimental animal infection unit (PFIE, Nouzilly, France).

#### Trial on phage effect on *Salmonella* burden

To evaluate phage effect on *Salmonella* burden, on the day of hatching, 80 broiler chicks were divided into two groups of 40 chicks. All 80 chicks were challenged by oral gavage with *Salmonella* Enteritidis LA5 at 5 × 10^4^ CFU/chick at Day 7. One group (“*S*E”) was not treated with phages. In the second group (“*S*E + Phages”), phages were continuously administrated to chicks via drinking water at a concentration of 10^9^ PFU/mL during their six first days of life (Day 1 – Day 6), then recurrently on days 8, 11, 15 and 18 (1, 4, 8 and 11 days post-infection respectively). On Days 1, 5, 7 and 14 post-infection, 8 chicken cecal contents per group were collected to quantify *Salmonella* levels. No significant difference in weight was detected between the two groups of animals.

#### Trial on fitness cost associated with partial resistant clone

Broiler chickens were also used to evaluate the fitness cost associated with a partial resistant clone by comparing its colonization ability in chickens to that of the *S*E reference strain. One hundred newly hatched chicks were divided into two groups of 50 chicks. At 7 days of age, one group (“*S*E”) was challenged by oral gavage with *Salmonella* Enteritidis LA5 at 5 × 10^4^ CFU/chick and the other group (“Partial resistant clone”) was challenged with the *Salmonella* partial resistant strain, ΔSE17-1, at 5 × 10^4^ CFU/chick. *Salmonella* growth was monitored in both groups on days 4, 7 and 14 post-infection by sampling 10 chicken cecal contents and fecal excretions per group and per day for bacterial enumeration.

### Statistical analysis

Statistical differences in the mean *Salmonella* counts (CFU/g) between in vivo groups were assessed using Wilcoxon-Mann–Whitney tests using R software (version 1.4–2). For this comparison a non-parametric permutational test, performed using the wilcoxon_test function of the R-package coin (v. 1.4–2, 10 000 permutations), yielded the same result. Unpaired *t*-test were used to test the difference between means of adsorption, burst size values and auto-aggregation rates using GraphPad Prism 6 software (GraphPad Software, USA).

### Data availability

The genome sequences of bacteria with long reads from MinION Oxford Nanopore and short reads from Illumina MiSeq sequencing technologies used in this study are available in the NCBI databank under the accession BioProject numbers PRJNA984603 and PRJNA984647 respectively.

## Results

### Effect of the phage cocktail on *Salmonella* burden

**Figure 1:**
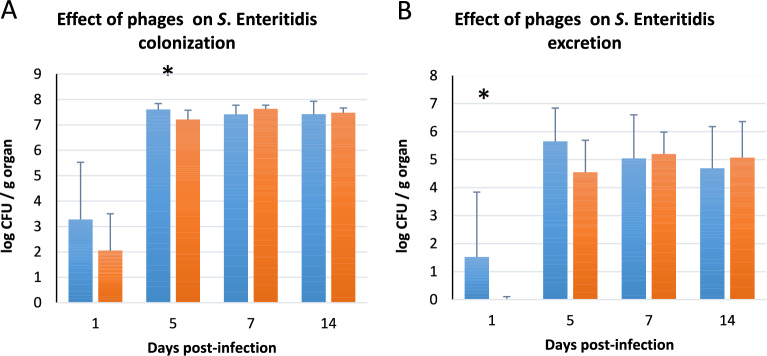
**Effect of the recurrent administration of phage cocktail on** ***S***. **Enteritidis colonization and excretion in broiler chickens**. *Salmonella* counts were monitored from the caeca (**A**) and the fecal samples (**B**) collected from the group challenged by 5 × 10^4^ CFU/chicks at 7 days of age (“SE”, blue bar) and from the group challenged by *Salmonella* and orally inoculated with phages at 10^9^ PFU/mL between days 1 and 6 and at 8, 11, 15, 18 days of age (“*S*E + Phages”, orange bar). Levels of *Salmonella* were expressed as mean log10 CFU per gram organ in 8 chicks per group. Statistical differences between the groups were calculated using the Wilcoxson test with * P < 0.05.

The effect of repeated phage administration on *Salmonella* load was first evaluated. For this purpose, two groups of chicks were challenged with 5 × 10^4^ CFU/chick of *Salmonella* Enteritidis LA5 (designated as *S*E reference strain) at 7 days of age (“*S*E” and “*S*E + Phages” groups). One of them (“*S*E + Phages” group) had received phages for the first 6 days of age through drinking water plus additional oral administration of phages on days 8, 11, 15, 18. This six-phage cocktail has already been used in a prophylactic trial [11]. The *Salmonella* load of “*S*E” group was compared to that of the “*S*E + Phages” group (Figure 1). Although an inhibitory effect was observed during the initial days following infection, it gradually diminished over time. This was observed both in fecal samples (with a significant drop at 1 day post-infection), and in ceca (with a significant drop at 5 days post-infection) (Figure 1). However, despite further phage administration, *Salmonella* colonization increased 7 days post-infection in “*S*E + Phages” group, reaching the level of *Salmonella* colonization in the “*S*E” group by the end of the experiment. Thus, the emergence of phage resistance by the *Salmonella* strain, which may explain the reduced efficacy of the phages during the trial, was investigated.

### Rate of bacteriophage-insensitive *Salmonella* clones

**Figure 2:**
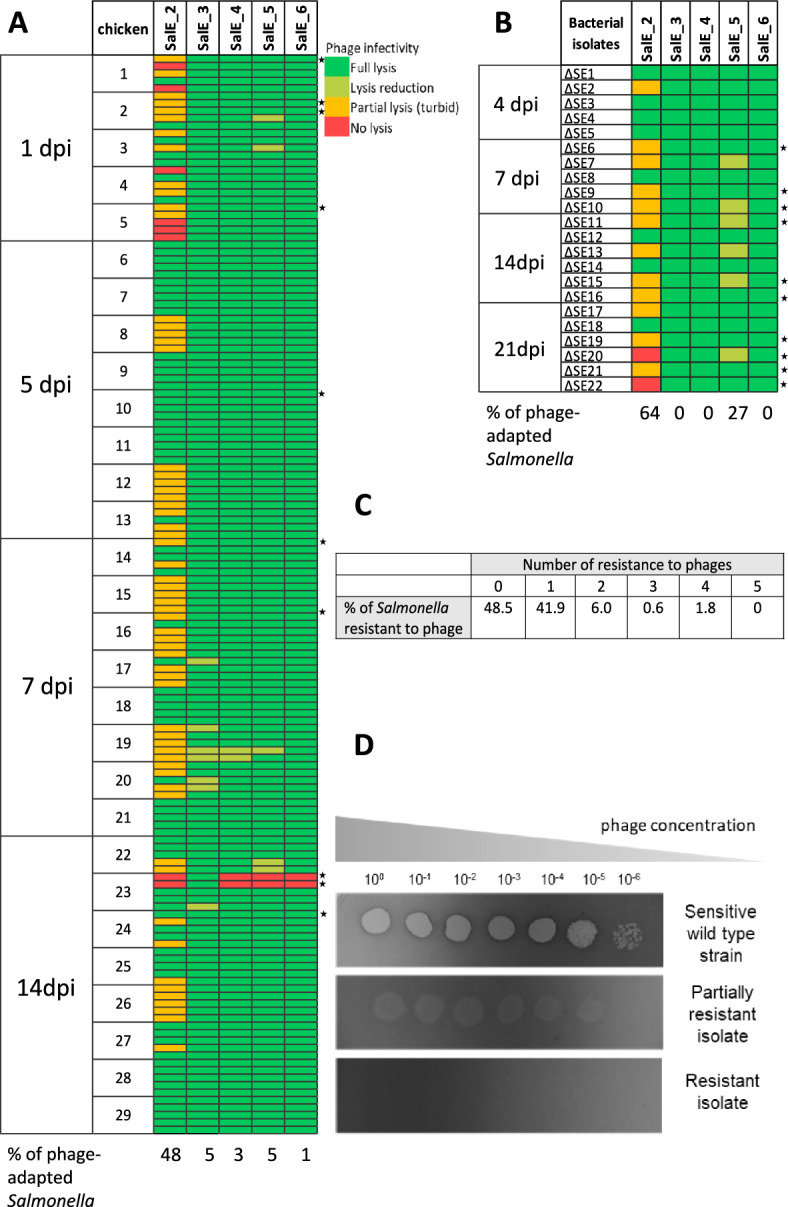
**Susceptibility** **towards the six phages of** ***Salmonella*** **isolates recovered from cecal contents of phage-treated chicks, at different days post-infection**. **A** A panel of 145 random isolates from 29 chicks (5 per chick) at 1, 5, 7 and 14 days post-infection was tested by spot assay. The infectivity pattern of each phage on these isolates was compared to their infectivity pattern on the *S*E reference strain. **B** A panel of 22 isolates recovered from an independent trial [11] performed with the same phage cocktail and the same *Salmonella* strain was also tested by spot assay. Phage activity profiles are represented by different colors as complete lysis (dark green) and reduction of the lysis (light green) for sensitive clones, partial lysis (orange) for partial resistant clones and no lysis (red) for complete resistant clones. Stars on the right represent the 20 clones of *Salmonella* screened for the presence of mutations by PCR. **C** The percentage of isolates susceptible to all phages or resistant to one or more phages was summarized. (**D**) Plaque morphology induced by the phage SalE_2 on lawns of the reference strain *S.* Enteritidis (wild type strain) and on lawns of isolated *Salmonella* clones, classified as partially resistant and fully resistant.

The phage susceptibility of *Salmonella* strains recovered from animals was tested against the six individual phages of the cocktail to determine whether the lack of protection was due to emergence of one or more resistances. A total of 145 random isolates were tested (Figure 2A) in addition to the 22 isolates recovered from the trial previously performed [11] with the same phage cocktail and the same *Salmonella* strain (Figure 2B). Most of the recovered clones were still sensitive to SalE_3, SalE_4, SalE_5, SalE_6 with only 5% of the bacterial isolates showing a slight reduction in their susceptibility to the SalE_3 and SalE_5. The main changes were observed in the susceptibility of *Salmonella* towards SalE_2. Indeed, around 6% of the total isolates were fully resistant and 42% were less susceptible (Figure 2). This predominantly observed phenotype of less susceptibility toward this phage was defined as partial resistance when phage titer was not reduced but the spot of bacteria was not completely cleared, leading to the formation of turbid spots (Figure 2D). This phenomenon suggested that *Salmonella* growth could occur despite the phage multiplication. Furthermore, both types of resistance (partial resistance, and complete resistance) were observed both after repeated phage administration and after prophylactic administration of phages to infected chicken. They were therefore the subject of further study. Importantly, while half the isolates were still susceptible to all phages, the remaining isolates were adapted to a single phage (42%) and only 8% were adapted to 2 or more phages (Figure 2C).

### In vitro stability of resistance phenotypes

**Figure 3:**
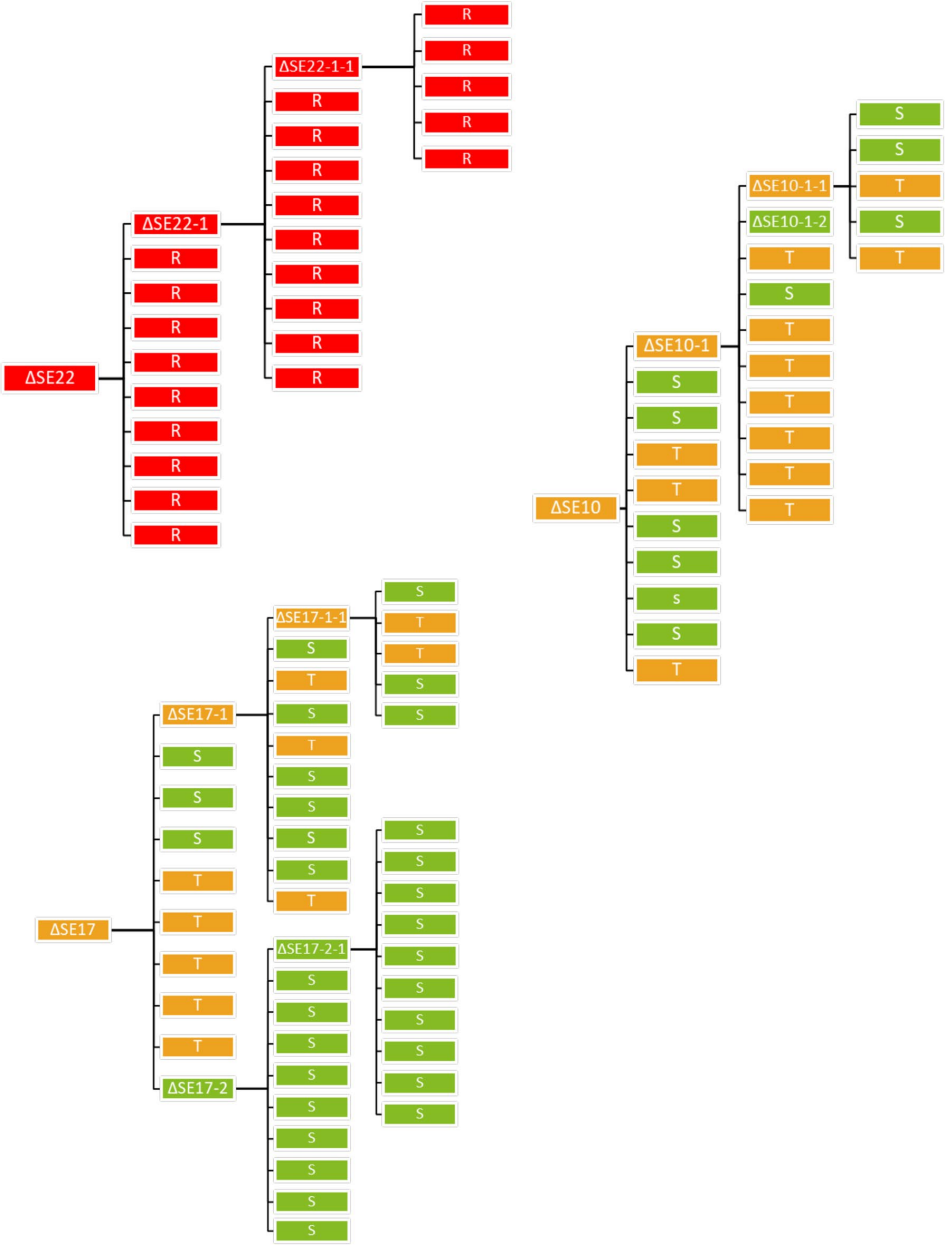
**Schematic representation of the in vitro stability of resistance phenotypes observed in ΔSE10, ΔSE17, and ΔSE22 isolates over three consecutive isolations and subcultures.** Bacterial clones were isolated and subcultured to generate bacterial lawns where spot assays were performed to test their susceptibility toward the SalE_2. This procedure was successively repeated 3 times (over 3 subcultures of daughter cells). The three phenotypes observed towards the studied phage were represented as sensitive by green squares, partial resistant by orange squares and complete resistant by red squares.

The stability of the different phenotypes of resistance observed towards SalE_2 (partial resistance and complete resistance) from clones recovered from chicken cecal content was evaluated. Three random isolates, one with complete resistance and two with partial resistance (ΔSE22, ΔSE10 and ΔSE17 respectively), were selected. They were cultured in vitro and sequentially isolated, in the absence of phage, three consecutive times to yield three subcultures of daughter cells. Following these sequential isolations, the daughter cells showed different phenotypes of resistance towards SalE_2 (Figure 3). The clone ΔSE22 stably retained complete phage resistance through the three subsequent isolations and subcultures. The partial resistance phenotype expressed by the clones ΔSE10 and ΔSE17 was, on the contrary, an unstable phenomenon. Indeed, for both clones (ΔSE10 and ΔSE17), 6/10 and 4/10 bacteria from the first isolation and subculture reverted to a sensitive phenotype respectively. Similarly, from the second isolation and subculture of these partial resistant clones (ΔSE10-1 and ΔSE17-1), 2/10 and 6/10 of their daughter cells reverted to a sensitive phenotype respectively. However, the sensitive clones subsequently generated by isolations and subcultures from the partial resistant clone (ΔSE17-2) were found to retain their phage susceptibility over the generations, when cultured in the absence of phage. Thus, *Salmonella* showed a reversible partial resistance acquisition, where variation of the phenotype occurs in switching from partial resistance to sensitivity. The three sequential isolations performed also suggest that the observed phenotypic heterogeneity of partial resistant clones was not due to the initial existence of a mix of bacterial populations.

Five of these generated clones, two sensitive clones ΔSE10-1–2, ΔSE17-2 and two partially resistant clones ΔSE10-1, ΔSE17-1 (see Figure 3), were randomly selected along with the one fully resistant clone ΔSE22, for further phenotypic and genetic analysis to decipher the mechanism of underlying resistance.

### Phage adsorption and one-step growth

**Figure 4:**
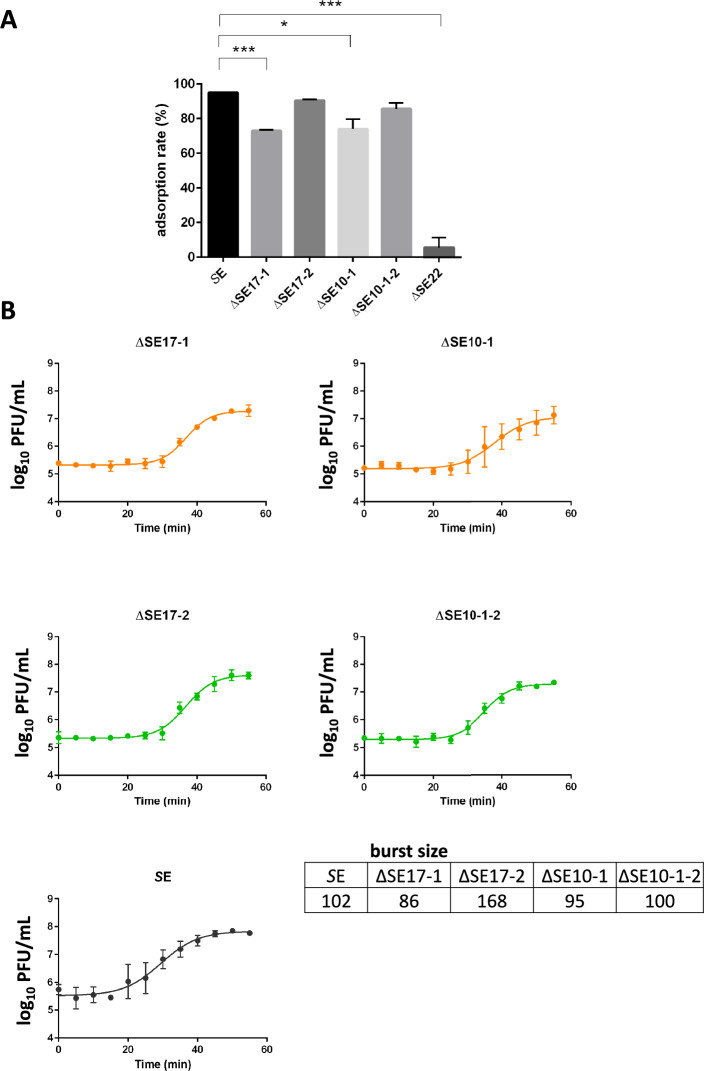
**Characterization of the different bacterial resistance phenotypes by analysis of the lytic activity of SalE_2.** Five clones recovered after in vivo infection were compared to the *S*E reference strain: two partial resistant clones (ΔSE17-1, ΔSE10-1), two sensitive clones (ΔSE17-2, ΔSE10-1–2), and one resistant clone (ΔSE22). **A** Absorption rate of the phage on the five different clones after 15 min of contact at an MOI of 0.1, 37 °C. **B** One step growth curves of SalE_2 on the different bacterial clones at an MOI of 0.1 Burst size was estimated by calculating the ratio of liberated phages to PFU enumerated prior to the onset of lysis and reported in a table. Phage replication dynamic on the ΔSE22 strain was not evaluated as SalE_2 does not replicate on this strain. Data represent the mean ± SD of three independent experiments. Statistical differences between adsorption rates and burst size values were calculated using *t*-tests. No statistical differences were found between burst sizes values.

Phage adsorption and one-step growth assays were performed to study SalE_2 lytic activity on the different bacterial resistance phenotypes. The percentage of phages able to adsorb onto the different bacterial clones was first determined (Figure 4A). The phage SalE_2 exhibited a reduced capacity to adsorb onto the two clones expressing the partial resistance phenotype. Specifically, the phage adsorption rate significantly decreased from 93% on the *S*E reference strain to 73% (*P* < 0.001) on the ΔSE17-1 clone and 74% (*P* = 0.02) on the ΔSE10-1 clone. SalE_2 exhibited a lack of adsorption onto the resistant clone ΔSE22, with the adsorption rate dropping to 2% (*P* < 0.001). In contrast, SalE_2 maintained a high adsorption rate of around 90% on the sensitive clones ΔSE17-2 and ΔSE10-1–2 (*P* > 0.05). Thus, the phenomenon of partial resistance appears to be associated with an adsorption deficiency, which reduced the ability of phages to infect *Salmonella*.

To determine whether phage replication rate could also be involved in the partial resistance phenotype, the replication dynamics of SalE_2 in clones were analyzed by one-step growth curves (Figure 4B). Phage replication dynamic in the ΔSE22 strain was not evaluated because SalE_2 does not adsorb onto it and therefore cannot infect it. The one-step growth curve of SalE_2 on the strains ΔSE17-1, ΔSE17-2, ΔSE10-1 and ΔSE10-1–2, showed a latent period of approximately 25 min while it was approximately 15 min on the *S*E reference strain. Regarding phage proliferation, the one-step growth curves showed a slight decrease on the partial resistant clones with burst sizes of 86 and 95 PFU/bacteria released by the ΔSE17-1 and ΔSE10-1 strains respectively compared to the 102 PFU/bacteria released by the reference strain. Nevertheless, these burst sizes were not statistically different, demonstrating that SalE_2 was also able to multiply in bacterial clones with partial resistance.

### Auto-aggregation assay tests

**Figure 5:**
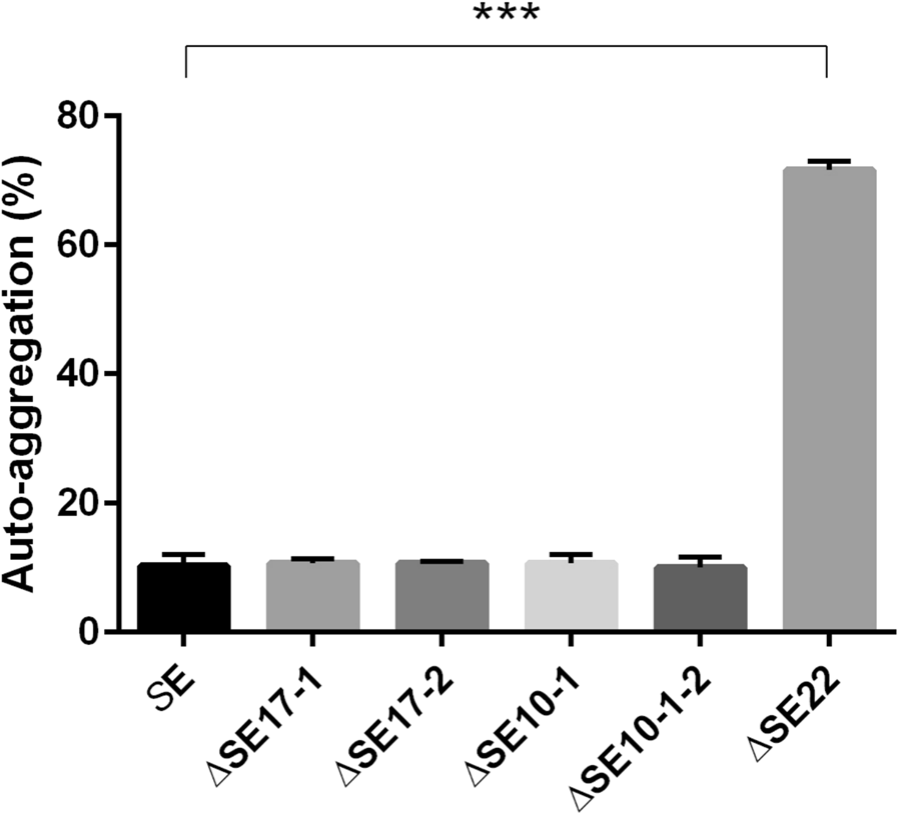
**Auto-aggregation rate of the different clones after static growth culture for 18 h at 37 °C. **OD_600_ values of the supernatant of the static cultures were measured from the five clones under scrutiny: two partial resistant clones (ΔSE17-1, ΔSE10-1), two sensitive clones (ΔSE17-2, ΔSE10-1–2), and one resistant clone (ΔSE22). Statistical differences were calculated using *t*-tests with *** P < 0.001. Data represent the mean ± SD of three independent experiments.

In addition to the lack of adsorption of the phage, the resistant clone ΔSE22 was observed to sediment during static liquid cultures (data not shown). Therefore, auto-aggregation tests were performed by measuring OD_600_ from the static cultures of the different clones (Figure 5). The percentage of aggregation was about 10–12% for the sensitive and partially resistant clones, compared to 10% for the initial strain *S*E. In contrast, the resistant clone ΔSE22 exhibited a significantly higher aggregation rate of 71% (*P* < 0.001). This phenotypic feature is typical of *Salmonella* strains exhibiting a rough phenotype and suggests an alteration in the LPS of this resistant clone.

### LPS structure analysis

**Figure 6:**
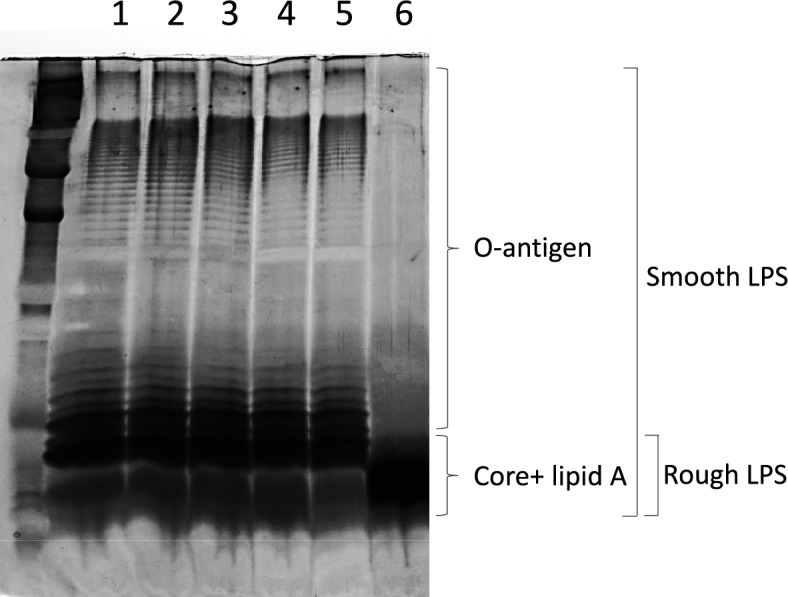
**SDS-PAGE analysis of the lipopolysaccharide (LPS) of different** ***Salmonella*** **clones compared to the** ***S*E reference strain**. Lipopolysaccharides (LPS) were separated on a 15% polyacrylamide gel stained with silver nitrate. Each band represents a repeat unit. Smooth LPS with O-antigen, core, and lipid A parts and rough LPS with truncated O-antigen are depicted on the right. Lane 1: *S*E LA5 wild type strain; Lane 2: ΔSE17-1; Lane 3: ΔSE17-2; Lane 4: ΔSE10-1; Lane 5: ΔSE10-1–2; Lane 6: ΔSE22.

The LPS O antigen repeat unit patterns of the five clones (the resistant ΔSE22 clone, the two partial resistant ΔSE17-1, ΔSE10-1 clones, and the two sensitive ΔSE17-2, ΔSE10-1–2 clones) were analyzed by SDS-PAGE after silver staining and then compared to the *S*E reference strain (Figure 6). The results showed no visible impact on the LPS profiles of the susceptible and partial resistant clones tested. Indeed, no differences were observed between the ΔSE17-1 and ΔSE10-1 clones compared to the *S*E reference strain or the susceptible clones. In contrast, the LPS profile of the ΔSE22 clone clearly showed deficiency with a loss of the O antigen and part of the core portion of the LPS compared to the reference strain. This incomplete LPS harboring a truncated structure confirms the rough phenotype of the strain.

To go further in the analysis of the LPS of the different clones, glycosyl composition and linkage analysis, as well as 1- and 2-dimensional NMR spectroscopy of O-specific polysaccharides of the clones were performed. Based on ^1^H and limited 2D NMR data analysis, all LPS samples contain a LPS identical to that of serogroup D1 (containing *S.* Enteritidis). Compositional and linkage results show Table 2 that all O antigen samples contain residues and linkages consistent with known *S.* Enteritidis LPS structures (2,3-Man, 4-Rha, 3-Gal, t-Tyv). Additionally, an increase in glucose levels was observed in clones recovered after infection compared with the inoculated strain, in both composition and linkage analyses. For example, samples from strains ΔSE17-1, ΔSE10-1 and ΔSE10-1–2 showed the highest amounts of terminal glucose (t-Glc), and samples ΔSE17-1 and ΔSE17-2 the highest amounts of 4-Glc. The increase in t-Glc correlated with a similar increase in 4-substituted galactose (3,4-Gal) in samples from ΔSE17-1, ΔSE10-1 and ΔSE10-1–2 strains (Table 2). This is consistent with some known structures of *Salmonella* O antigen, said to be glucosylated, where t-Glc is attached to galactose in position 4. Conversely, in these strains, notably in the partially resistant clones ΔSE17-1 and ΔSE10-1, the percentage of 3-Gal, representing unsubstituted galactose residues, is the lowest among the samples tested. Thus, the presence of glucosylated O antigen correlates with partial resistance without branching specificity.

**Table 2:** **Relative percent area of the PMAAs detected with the** ***Salmonella*** clones*

Sample	SE	∆SE17-1	∆SE17-2	∆SE10-1	∆SE10-1–2
Residue	Percent Area				
t-Tyv	7.9	6.4	4.6	5.9	8.6
2-Rha	0.8	0.4	0.2	0.4	0.4
t-Man	0.3	0.4	0.2	0.3	0.4
4-Rha	24.6	19.6	18.0	21.2	22.8
t-Glc	0.8	8.3	2.2	13.1	12.6
t-Gal	1.6	1.1	1.0	1.3	0.9
3,4-Rha	0.5	0.4	0.3	0.5	0.3
2,4-Rha	0.6	0.4	0.3	0.6	0.4
2-Man	1.2	1.0	1.4	1.0	1.0
3-Man	7.7	4.8	6.1	4.9	5.7
2,3,4-Rha	0.1	0.2	0.2	0.3	0.3
t-Hep	1.0	1.0	0.6	0.8	0.5
3-Gal	29.3	15.4	21.8	13.1	16.2
2-Gal	0.8	0.7	0.6	0.9	0.6
4-Glc	0.4	12.2	19.0	1.0	0.8
2,3-Man	18.5	15.3	15.1	16.9	16.3
3,4-Gal	1.7	7.8	1.2	12.9	11.4
2,4-Man	0.7	1.1	0.9	1.0	n.d
3,6-Man	0.8	0.9	0.6	1.0	0.5
4,6-Glc	0.04	1.3	2.4	0.3	0.1
3,6-Gal	0.3	0.3	0.2	0.3	n.d
3,4,6-Man	n.d	0.3	0.1	0.3	0.2
3,4,6-Glc	0.1	0.1	0.1	0.3	n.d
2,3,6-Man	0.1	0.3	0.1	0.5	0.1
2,3,4,6-Man	n.d	0.2	0.0	0.4	n.d
2,3,4,6-Gal	n.d	0.1	0.2	0.5	n.d
2,3,4,6-Glc	n.d	0.2	2.5	0.4	n.d

n.d. – not detected

^*^Glycosyl linkage analysis was performed by combined gas chromatography–mass spectrometry of the partially methylated alditol acetates (PMAAs) derivatives produced from the *Salmonella* clones. The procedure is a slight modification of the one described by Heiss et al. [29].

### Whole genome sequencing and mutations identification

The five clones under scrutiny were subjected to whole-genome sequencing. The identified genetic variations were then analyzed to determine potential involvement in the observed phage resistance phenotypes. The genome of the *S*E reference strain was used as a reference genome to identify mutations. Few mutations were found in the genomes of the different clones as compared to the reference strain *S*E (Table 3). Overall, three types of genetic variations were identified by SNP analysis. However, both couples of progeny clones (sensitive and partial resistant clones) coming from the same clone were found to share identical mutations even though their expressed phenotypes were different. As no common mutations were found between the partial resistant clones, this analysis confirmed that the genetic variations were not at the origin of the partial resistance phenotype observed. On the clones ΔSE17-1 and ΔSE17-2, a nonsense mutation occurred in the *motB* gene which produced a stop codon instead of a glutamine amino acid resulting in gene termination and truncation of the encoding protein. The *motB* gene is a flagella related gene involved in motive force for flagellar rotation. On the clones ΔSE10-1 and ΔSE10-1–2, a synonymous mutation occurred in the middle of *nlpD* open reading frame (which encodes a lipoprotein associated with the outer membrane). This mutation is localized before the transcription starting site of *rpoS*, whose promotor  is located in the *nlpD* open reading frame. Consequently, if the synonymous mutation cannot altered the role of the NlpD protein in the outer membrane, we cannot exclude that this mutation altered the transcription of *rpoS*, which plays a critical role in regulating the bacterial general stress response. On the resistant clone ΔSE22, there was a deletion of 29 nucleotides in the *rfbD* gene at the 754th nucleotide position of the gene, resulting in a frameshift. This gene encodes a dTDP-4-Dehydrorhamnose reductase and is involved in the O-antigen biosynthesis. The mutation observed would result in the disruption of LPS synthesis and could explain the truncation and lack of the O-antigen and the core part of the LPS. No other mutations were identified on the virulence plasmid.

**Table 3:** **SNP, insertion or deletion detected in the genome of five selected isolated clones compared to the** ***S*E reference strain**

Strain	Mutation type	Nucleotide change (position)	Amino acid change	NT position on the gene/ gene length (bp)	Affected gene	Effects
∆SE17-1	SNP	G → A 2,744,155	Gln → STOP	454/ 930	*motB*	nonsense → truncated protein
∆SE17-2	SNP	G → A 2,744,155	Gln → STOP	454/ 930	*motB*	nonsense → truncated protein
∆SE10-1	SNP	C → G 967,887	Gly → Gly	351/ 855	*nlpD* *rpoS?*	Synonymous Undetermined
∆SE10-1–2	SNP	C → G 967,887	Gly → Gly	351/ 855	*nlpD* *rpoS?*	Synonymous Undetermined
∆SE22	deletion	C → 29nucl 1,738,169		756–784/ 900	*rfbD*	Frameshift

Two partially resistant clones (ΔSE17-1, ΔSE10-1), two sensitive clones (ΔSE17-2, ΔSE10-1–2) and one completely resistant clone (ΔSE22) to SalE_2 were purified after in vivo infection and subsequently sequenced. Sequences were compared with the sequence of the inoculated strain

To assess whether the identified mutations were commonly found in the trials performed, the frequency of occurrence of these mutations was investigated by PCR and subsequent Sanger sequencing verification. Twenty clones of *Salmonella* (10 belonging to the first trial [11] and 10 to the current trial study as indicated in Figure 2) were screened for the presence of these 3 mutations. Results indicated that the majority of the partial phage resistant clones did not carry the mutations previously observed in the *motB* and *nlpD* genes. No additional clones carried the mutation in the *motB* gene and 3 out of the 14 additional clones tested carried the *nlpD* gene mutation. This result further suggests that genetic variations are not involved in the observed partial resistance phenotype and, therefore, no further investigations on *motB* and *nlpD* mutations were performed. On the contrary, two out of the four additional phage resistant clones tested carried the *rfbD* mutation as did the ΔSE22 resistant clone.

### Fitness cost associated with partial resistant clone

**Figure 7:**
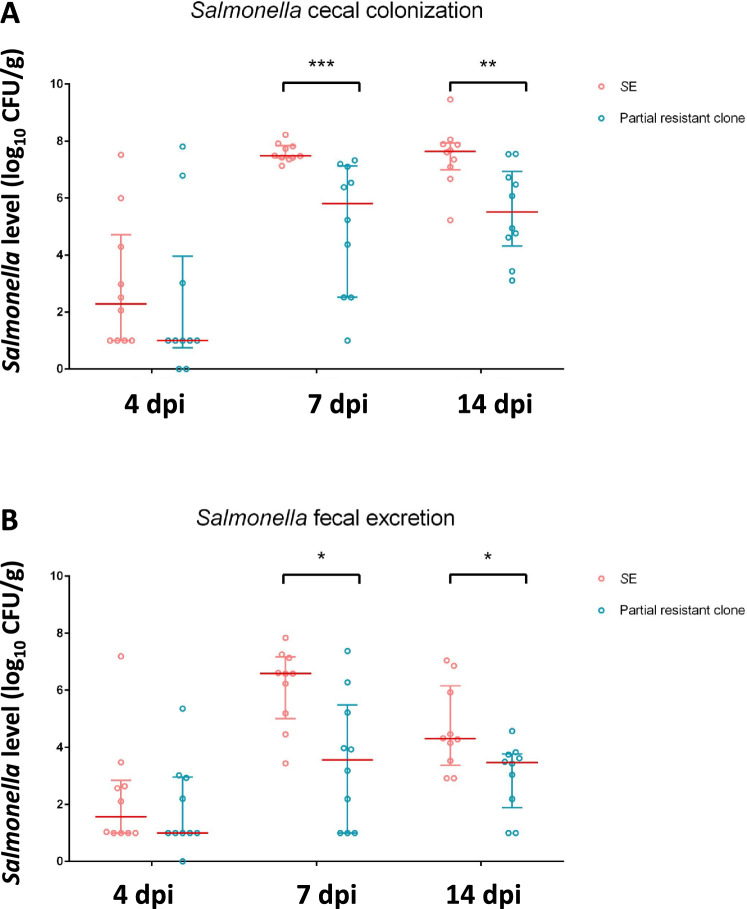
**Ability to colonize the chicken gut of a partial resistant** ***Salmonella* clone (ΔSE17-1) compared to the ability of the** ***S*E reference strain after 4, 7 and 14 days of infection.** ***Salmonella*** count was monitored from (**A**) cecal contents and (**B**) feces collected from the groups challenged at 7 days of age by 5.10^4^ CFU/chick of *S*E reference strain (“*S*E”) represented by pink circles for each animal and challenged with the same dose of ΔSE17-1 (“Partial resistant clone”) represented by blue circles for each animal. *Salmonella* was enumerated as CFU per gram of organ in 10 chicks per group. The red bars represent the median of *Salmonella* levels. Statistical differences between groups were calculated using the Wilcoxson test with * P < 0.05.

Emergence of bacterial resistance is often associated with a fitness cost, such as reduced virulence or impaired colonization, particularly when resistance affects receptors crucial for virulence, like LPS. *Salmonella* strains with mutations in the *rfbD* gene have been shown to exhibit increased sensitivity to environmental factors (pH, temperature, and antibiotic) [13]. Additionally, strains with a “rough” LPS phenotype are less able to colonize the gut and are less virulent [34–36]. Given these factors, the gut colonization ability of the resistant clone ΔSE22 was not investigated in this study. The focus was therefore on the ability of partially resistant clones to colonize the chicken gut. Newly hatched chicks were divided into two groups of 40 chicks that were challenged by 5 × 10^4^ CFU/chicks of either the wild type *Salmonella* Enteritidis LA5 strain or the randomly selected partial resistant clones (ΔSE17-1). *Salmonella* cecal colonization and fecal shedding were monitored for 21 days. The results showed that over the course of the experiment, the *Salmonella* colonization rate was lower in the group infected with the partially resistant clone compared to the control strain (Figure 7). Indeed, cecal *Salmonella* loads were significantly reduced in the group infected with the ΔSE17-1 clone, by an average of 1.1 log_10_ (*P* < 0.001) at 7 days post-infection and by 2.7 log_10_ (*P* = 0.003) at 14 days post-infection (Figure 7A). Similarly, fecal *Salmonella* excretions were significantly reduced by 2.6 log_10_ (*P* = 0.017) and 0.8 log_10_ (*P* = 0.037), 7 and 14 days post-infection, respectively (Figure 7B). While the reduction of *Salmonella* 4 days post-infection was not statistically significant, the cecal and fecal colonization of the ΔSE17-1 clone were 1.3 log_10_ and 0.6 log_10_ lower respectively compared to the SE strain. These results indicate a colonization deficiency associated with the ΔSE17-1 clone, suggesting that phage-driven adaptation has impaired virulence of the strain. Furthermore, the observation that only 75% of the *Salmonella* clones recovered at 14 days post-infection retained the partial resistance phenotype supports the hypothesis that this trait is reversible in both in vitro and in vivo conditions.

## Discussion

Bacterial adaptation to phage predation is a natural mechanism that occurs over the course of phage-bacteria interactions in the environment. However, the possible deployment of bacterial defense mechanisms toward phage attack is challenging for phage therapy development.

A first study evaluated the prophylactic efficacy of a phage cocktail and demonstrated its ability to reduce the *Salmonella* load during the early stages of poultry production. To improve the therapeutic efficacy of the same phage cocktail, the therapeutic effect of a recurrent phage administration was tested. However, this study showed that recurrent administration of a cocktail containing six phages leads to adaptation of *Salmonella* to a single phage from the cocktail, through two distinct resistance patterns. The first resistance pattern exhibited a complete lack of phage adsorption, suggesting either the loss or inaccessibility of the receptor. Comparative genomic and phenotypic analyses revealed that the phage resistant clone carried a 29-nucleotide deletion in the *rfbD* gene at the 754th nucleotide position of the gene, causing a frameshift mutation, which led to the loss of the LPS O-antigen and part of the core region. The *rfbD* gene encodes a dTDP-4-Dehydrorhamnose reductase, an essential enzyme for rhamnose synthesis that plays a role in LPS O-antigen biosynthesis. Therefore, inactivation of this gene likely led to the absence of the O antigen giving the resistant clone a rough LPS phenotype [37–39]. The inability of phage SalE_2 to form plaques on this resistant clone suggests that the depleted portion of the LPS is necessary for SalE_2 infection. Based on these findings, we propose that the LPS O-antigen or a part of the core region serves as the primary receptor for SalE_2. This aligns with previous studies showing that many *Salmonella*-infecting phages use LPS components as primary receptors, as LPS is a major structural component of the bacterial outer membrane. This makes LPS modifications a common strategy employed by bacteria to evade phage infection [40]. The exploitation of LPS as a receptor through *rfb* genes is observed for other *Salmonella* phages such as phage P22 as well as P6, Reaper_GE and phage S55 where *rfb* genes are required for adsorption process [41–43]. While LPS modifications often arise from spontaneous genetic mutations [13, 41–44], in some cases, bacterial surface modifications occur through non-genetic mechanisms.

While the first resistance pattern demonstrated a stable genetic inheritance of the resistant phenotype across multiple generations of subpopulations, the second pattern exhibited a distinct behavior, characterized by partial and transient resistance to phages. Specifically, the emergence of partial resistance to phages was characterized by the formation of turbid plaques, indicating incomplete lysis of the bacterial population. Similarly, Hurley et al. observed the emergence of partial resistance with the formation of hazy plaques following repeated phage administration in chickens infected with *Salmonella* Enteritidis [45]. In conjunction with this specific pattern, a slight deficit in phage adsorption was observed, but no genetic mutations were found to explain this phenotype, suggesting a phenotypic resistance rather than a genetic basis for resistance [46]. Variations in bacterial gene expression could lead to modifications of bacterial surfaces receptors, potentially explaining the observed population heterogeneity within a genetically uniform colony, as evidenced by the formation of turbid plaques. Furthermore, the subpopulations of clones generated exhibited transient partial resistance. Examples of this strategy have been described in *Salmonella*, where bacteria can transiently resist phage infection by regulating, activating, or deactivating various functional genes in response to phage exposure without inducing mutations in these genes [47]. In this study, we showed that partial resistance correlates with the presence of glucosylated O antigen. This result supports the initial observation that SalE_2 uses LPS as a receptor. The phenotypic heterogeneity within the population, along with the observation that clonal bacteria recovered after infection revert to a phage-sensitive phenotype suggests a phase variation process, which refers to the heritable but reversible process of ON–OFF switching of gene expression, leading to the expression or non-expression of specific phenotypes [48, 49]. In line with this result, we can note that the *Salmonella* glycosyltransferase operon (gtr), which is able to modify the O antigen, is regulated by phase variation [50]. Moreover, phase variation and LPS modifications are involved in phage resistance mechanisms [51]. This adaptive strategy allows *Salmonella* to maintain its population, while the phage persists by multiplying on sensitive clones, as shown in this study and that of Shkoporov et al*.* [52]. This co-existence phenomenon was also demonstrated by the long-term persistence of SalE_2 in the gut during the in vivo experiment, in parallel with the persistence and growth of *Salmonella* Enteritidis [11].

Modification of its LPS can therefore enable the bacterium to resist destruction by phages. However, as LPS plays a critical role in bacterial infection, previous studies have demonstrated that *Salmonella* strains with a rough phenotype are avirulent in vivo [34–36]. This is the case for strains having a mutation in the *rfb* genes as observed in our condition [53–57]. Our results support the idea that removing the O-antigen by mutation in the *rfbD* gene can have a significant impact on bacterial fitness as we did not observe a substantial population of this phage-resistant clone persisting in the chicken gut. Thus, while complete resistance carries a significant fitness cost for the bacterium, the fitness consequences of partial resistance are likely to be less severe due to its partial and transient nature. Nevertheless, even under these conditions, our results showed a reduced ability of the partially resistant clone to colonize the gut. It should be noted that the presence of a *motB* gene mutation in the clone used could also have an impact on its colonization abilities. However, this effect is unlikely to be significant, as studies by Barbosa et al. demonstrated that loss of motility in flagellated strains carrying a mutation in the *motB* gene (SEΔmotB) did not affect fecal shedding of *Salmonella* Enteritidis in chickens [58]. Furthermore, they reported that the ability of SEΔmotB to colonize the ceca was only slightly affected in the early stages of chicken infection (up to 5 days post-infection) and was unaffected thereafter.

In summary, our study has shed light on potential adaptive mechanisms of *Salmonella* following in vivo phage treatment, which is crucial for the further development of phage therapy. Notably, our study shows that *Salmonella* had mostly adapted to a single phage from the cocktail, with no detectable cross-resistance. This suggests that the phage SalE_2 utilizes a distinct bacterial entry mechanism compared to the other phages in the cocktail. The limited protective efficacy observed with recurrent phage inoculation compared to prophylactic treatment, cannot be attributed to insufficient in vivo persistence of the phages, as was previously suggested in the case of prophylactic inoculation [11]. In this experiment, phages were present throughout the experiment, ruling out inadequate phage persistence as the primary factor. Instead, the performance of individual phages appears to play a more critical role. The fact that SalE_2 has a shorter latency period and a higher burst size as previously described [11] suggests that it may outcompete other phages in infecting the host bacterium without killing the host. Interestingly, our in vitro assays revealed that co-inoculation of SalE_2 with *S.* Enteritidis initially reduced bacterial counts, followed by a resurgence of both phage and bacterial populations. This pattern is coherent with the development of partial resistance, potentially enabling a dynamic equilibrium where the phage persists without eliminating the bacterial host. This could explain the predominance of partially resistant *Salmonella* isolates to SalE_2 in our study. It could be thus interesting to test the cocktail without the SalE_2 phage.

Consequently, these findings underscore the need to optimize the phage cocktail to ensure effective protection against *Salmonella* infection in poultry. While it is well established that therapeutic phage cocktail must be capable of targeting diverse bacterial strains, maintaining persistence, and limiting resistance development, our results emphasize an additional key factor: balanced efficacy, comparable in vivo stability and activity, and ideally, act synergistically to prevent the selection of escape mutants. Importantly, we show the importance of studying bacterial adaptation mechanisms, such as LPS modifications, which were associated here with reduced virulence. Rationally selecting phages that promote resistance with a fitness or virulence cost represents a promising strategy for phage cocktail design. By doing so, the evolutionary pressure exerted by the phage can be leveraged not only to control bacterial populations but also to attenuate their pathogenic potential. Thus, what is typically viewed as a drawback of phage resistance could be repurposed into a functional asset in phage cocktail design.
